# Chorein addiction in VPS13A overexpressing rhabdomyosarcoma cells

**DOI:** 10.18632/oncotarget.3582

**Published:** 2015-03-14

**Authors:** Sabina Honisch, Willi Yu, Guilai Liu, Ioana Alesutan, Syeda T. Towhid, Anna Tsapara, Sabine Schleicher, Rupert Handgretinger, Christos Stournaras, Florian Lang

**Affiliations:** ^1^ Department of Physiology, University of Tübingen, Tübingen, Germany; ^2^ Department of Biochemistry, University of Crete Medical School, Heraklion, Greece; ^3^ Department of Hematology and Oncology, Children's Hospital, University Hospital of Tuebingen, Tübingen, Germany

**Keywords:** cancer, apoptosis, PI-3K, BCL-2, bax

## Abstract

Chorein encoded by VPS13A (vacuolar protein sorting-associated protein 13A) is defective in chorea-acanthocytosis. Chorein fosters neuronal cell survival, cortical actin polymerization and cell stiffness. In view of its anti-apoptotic effect in neurons, we explored whether chorein is expressed in cancer cells and influences cancer cell survival. RT-PCR was employed to determine transcript levels, specific siRNA to silence chorein, FACS analysis to follow apoptosis and Western blotting to quantify protein abundance. Chorein transcripts were detected in various cancer cell types. The mRNA coding for chorein and chorein protein were most abundant in drug resistant, poorly differentiated human rhabdomyosarcoma cells. Chorein silencing significantly reduced the ratio of phosphorylated (and thus activated) to total phosphoinositide 3 kinase (PI-3K), pointing to inactivation of this crucial pro-survival signaling molecule. Moreover, chorein silencing diminished transcript levels and protein expression of anti-apoptotic BCL-2 and enhanced transcript levels of pro-apoptotic Bax. Silencing of chorein in rhabdomyosarcoma cells was followed by mitochondrial depolarization, caspase 3 activation and stimulation of early and late apoptosis. In conclusion, chorein is expressed in various cancer cells. In cells with high chorein expression levels chorein silencing promotes apoptotic cell death, an effect paralleled by down-regulation of PI-3K activity and BCL-2/Bax expression ratio.

## INTRODUCTION

Chorein is a powerful regulator of cytoskeletal architecture and cell survival [1]. The protein interacts with phosphoinositide-3-kinase (PI3K)-p85-subunit and probably sensitizes the PI3K-p85-subunit to tyrosine phosphorylation [1-3]. Presumably by up-regulation of PI3K activity chorein leads to activation of Rac1 and PAK1, triggers polymerization of cortical actin, fosters phosphorylation of Bad, and counteracts mitochondrial depolarization [1]. Loss-of-function mutations of VPS13A (vacuolar protein sorting-associated protein 13A), the gene encoding chorein, lead to chorea-acanthocytosis (CA), an autosomal recessive genetic disease [4-9] characterized by progressive hyperkinetic movement disorder, cognitive dysfunction, behavioral abnormalities, chronic hyperkalemia and variable acanthocytosis of red blood cells [5, 10]. Gene-targeted mice lacking chorein display erythrocyte shape changes [11], apoptosis of neurons [12] and behavioral abnormalities [12].

Chorein expression is not restricted to brain and erythrocytes, but is observed in diverse further tissues [13-15]. Chorein in blood platelets participates in the regulation of secretion and aggregation [15]. Moreover, chorein expressed in endothelial cells contributes to the regulation of endothelial cell stiffness [14]. Clearly, much is to be learned on the functional significance of this protein.

In view of the anti-apoptotic effect of chorein, we explored whether chorein is expressed in tumor cells and, if so, whether it modifies the survival of those cells. To this end, the chorein transcript and protein levels were determined in several tumor cells. As a result, chorein is expressed in some tumor cells with highest transcript levels found in ZF rhabdomyosarcoma cells. The impact of chorein on the survival of those cells was elucidated by chorein silencing and subsequent analysis of apoptosis and apoptotic signaling.

## RESULTS

**Figure 1:**
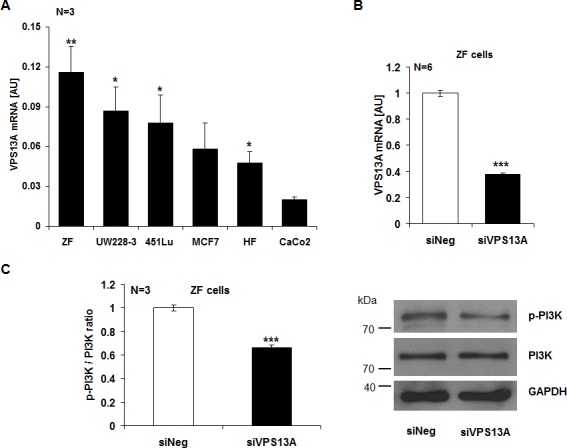
Chorein expression in tumor cells and chorein sensitive PI-3K phosphorylation in ZF rhabdomyosarcoma cells A. Transcript levels of chorein in various cell lines (ZF= rhabdomyosarcoma, UW228-3 = medulloblastoma, 451Lu= melanoma, MCF7= breast cancer, HF= human dermal fibroblasts, CaCo2= colon carcinoma). The chorein transcript levels were highest in drug resistant, poorly differentiated ZF rhabdomyosarcoma cells. *(p<0.05), **(p<0.01) significant difference to the CaCo2 cells. B. Efficiency of 48h chorein silencing. Chorein (VPS13A) mRNA levels were analysed by quantitative real-time PCR. Bars (A and B) indicate the mean values of 2^−ΔCt^ using GAPDH as housekeeping gene ± SEM from n=6 independent experiments. *** (p< 0,001; unpaired t-test) indicates significant difference to the negative silenced control (siNeg). C. Left: arithmetic means ± SEM (n=3) of the ratio of phosphorylated PI-3K p85 subunit to total PI-3K (p-PI-3K/PI-3K ratio) in ZF cells transfected with control siRNA (siNeg) and siRNA for chorein (siVPS13A). ***significant difference (p<0.001, unpaired t-test). Right: representative original western blots showing the protein abundance of phosphorylated (p85) PI-3K, total PI-3K and respective GAPDH as loading control.

**Figure 2:**
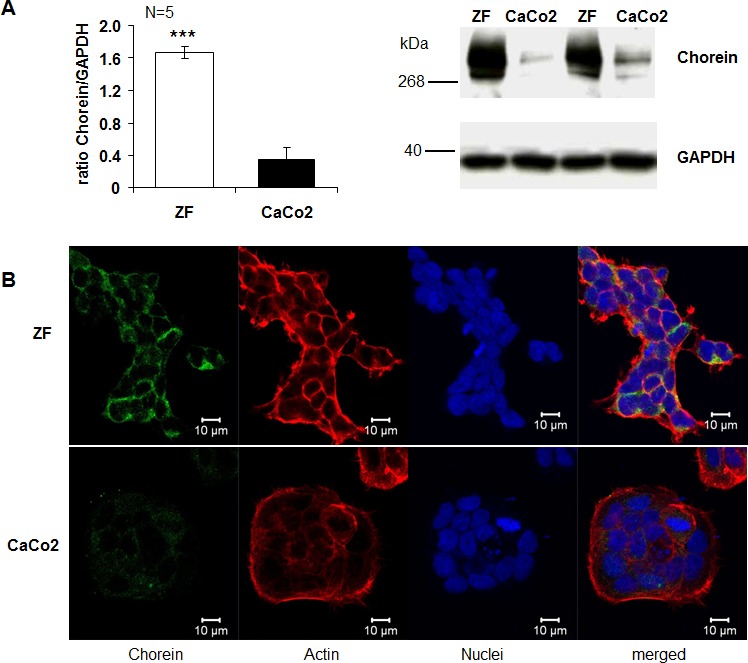
Chorein protein expression in ZF rhabdomyosarcoma cells and CaCo2 cells A. Left: arithmetic means ± SEM (n=5) of the ratio of chorein (VPS13A) to GAPDH in ZF cells (white bar) and CaCo2 cells (black bar) Right: representative original western blots showing the protein abundance of chorein and respective GAPDH bands. *** (p< 0,001; unpaired t-test) significant difference between ZF and CaCo2 cells. B. Confocal images of human rhabdomyosarcoma ZF cells (upper panel) and CaCo2 cells (lower panel) stained with anti-chorein antibody (green), rhodamine-phalloidin binding to F-actin (red) and DRAQ-5 for nuclei (blue).

The present study explored whether chorein is expressed in tumor cells and impacts on the survival of those cells. As illustrated in Fig. 1A, chorein is expressed in several tumor cell lines. mRNA coding for chorein was predominantly transcribed in drug resistant, poorly differentiated ZF rhabdomyosarcoma cells (Fig. 1A). Other cancer cell lines such as the human colon carcinoma CaCo2 cells expressed significantly lower levels of chorein (Fig. 1A). The differences in chorein transcript levels between ZF rhabdomyosarcoma cells and CaCo2 cells were paralleled by similar differences in protein expression. Accordimg to both, Western-blot and confocal scanning analysis chorein protein expression was markedly higher in ZF rhabdomyosarcoma cells than in CaCo2 cells (Fig, 2). According to confocal microscopy chorein protein is mainly localized in the cytoplasm (Fig. 2B).

In order to determine the functional significance of chorein, the chorein transcription has been suppressed by silencing. The silencing efficacy is shown in Fig. 1A, Suppl. Figs 1A and 2A. Fig. 1C illustrates the impact of chorein silencing on PI-3K phosphorylation. In ZF rhabdomyosarcoma cells chorein silencing decreased the ratio of phosphorylated to total PI-3K significantly, implying chorein sensitive phosphorylation of this crucial pro-survival signaling molecule. For comparison, chorein silencing had little effect on PI-3K phosphorylation in CaCo2 cells (Suppl. Fig. 1B).

**Figure 3:**
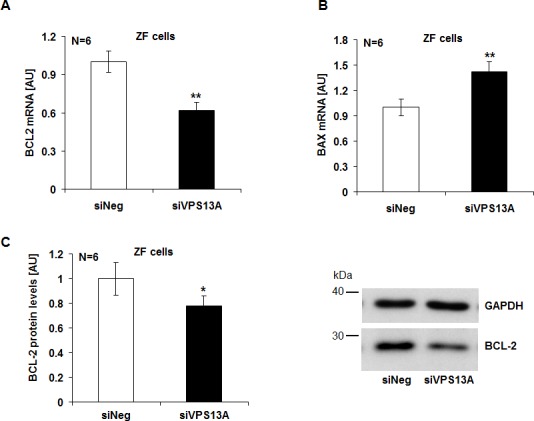
Chorein sensitive BCL-2 and Bax gene transcription and protein expression in ZF rhabdomyosarcoma cells Arithmetic means ± SEM (n=6) of the BCL-2 (A) and Bax (B) mRNA levels relative to GAPDH mRNA levels in ZF cells transfected with control siRNA (siNeg) or siRNA for chorein (siVPS13A). ** significant difference (p<0.01; unpaired t-test) C. Left: Arithmetic means ± SEM (n=6) of the BCL-2 protein levels compared to GAPDH levels in ZF cells transfected with control siRNA (siNeg) and siRNA for chorein (siVPS13A). Right: original Western blots of BCL-2 and GAPDH as loading control in ZF cells transfected with control siRNA (siNeg) or siRNA for chorein (siVPS13A). * significant difference (p<0.05; unpaired t-test).

Additional experiments addressed the impact of chorein on the expression of anti-apoptotic protein B-cell lymphoma 2 (BCL-2) and of pro-apoptotic protein Bax. As illustrated in Fig. 3A, chorein silencing significantly decreased the BCL-2 (Fig. 3A) and increased the Bax (Fig. 3B) transcript levels in ZF rhabdomyosarcoma cells. Furthermore, chorein silencing decreased the BCL-2 protein abundance in ZF rhabdomyosarcoma cells (Fig. 3C).

**Figure 4:**
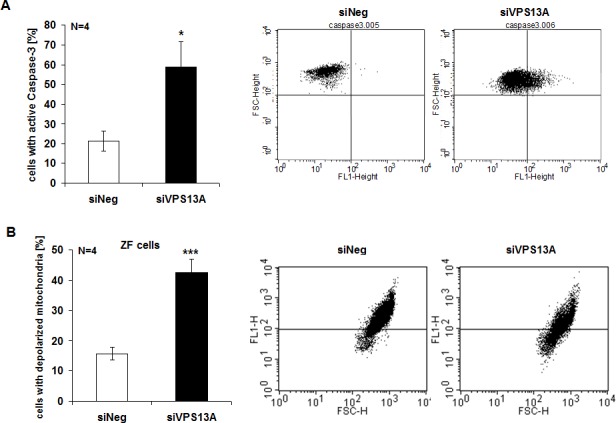
Chorein sensitive Caspase-3 activity and mitochondrial depolarization in ZF rhabdomyosarcoma cells A. Cells were stained with conjugated inhibitor of active Caspase-3 (FITC-DEVD-FMK) and measured by FACS. Shown are arithmetic means (left) ± SE (n=4) and representative original histograms (right) demonstrating caspase activity in ZF cells transfected with control siRNA (siNeg) or siRNA for chorein (siVPS13A). * significant difference (p<0.05; unpaired t-test). B. Arithmetic means (left) ± SEM (n=4) and representative original histograms (right) of mitochondrial depolarization measured by FACS in ZF cells transfected with negative control siRNA (siNeg) or siRNA for chorein (siVPS13A).*** indicates significant difference (p<0.001, unpaired t-test).

Caspase 3 activity was determined to elucidate whether chorein influenced apoptotic signaling. As illustrated in Fig. 4A, chorein silencing significantly enhanced caspase-3 activity in ZF rhabdomyosarcoma cells, an observation pointing to triggering of apoptosis. In line with this observation, chorein silencing of ZF rhabdomyosarcoma cells was followed by significant mitochondrial depolarization (Fig. 4B). Mitochondrial depolarization reached statistical significance already 24 h after chorein silencing and persisted throughout 72 h (Suppl. Fig. 2C).

**Figure 5:**
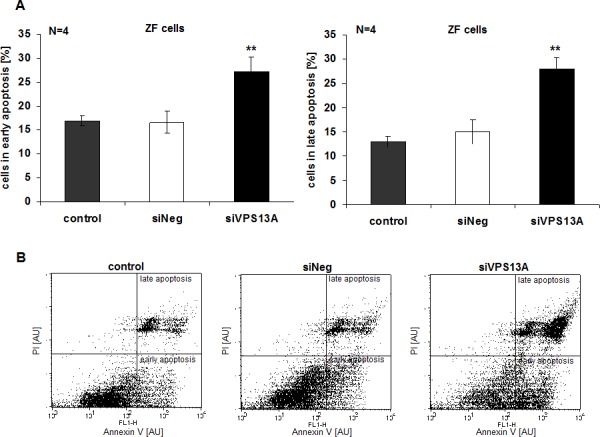
Chorein sensitivity of apoptosis in ZF rhabdomyosarcoma cells A. ZF cells were stained with FITC conjugated Annexin V and propidium iodide (PI). Presented are arithmetic means ± SEM (n=4) of early (left) and late (right) apoptosis in untransfected (control), transfected with negative control siRNA (siNeg) and with siRNA for chorein (siVPS13A) ZF cells. **significant difference (p<0.01; unpaired t-test) to the negative silenced cells (siNeg). B. Original dot-plots (PI/Annexin V) of a representative experiment.

**Figure 6:**
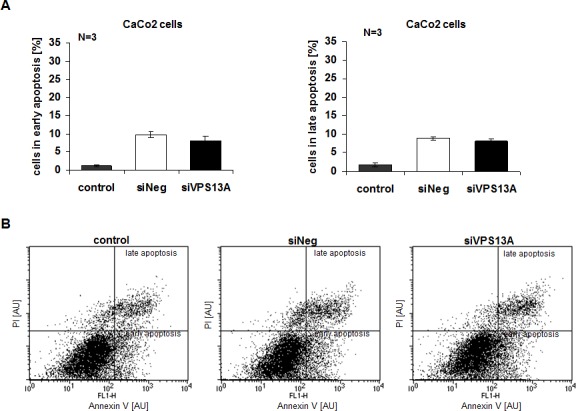
Resistance of CaCo2 cells to apoptotic effect of chorein silencing A. CaCo2 cells were stained with FITC conjugated Annexin V and propidium iodide (PI) and measured by FACS. Shown are arithmetic means ± SEM (n=3) of early (left) and late (right) apoptosis in non-transfected (control), transfected with negative control siRNA (siNeg) and with siRNA for chorein (siVPS13A) CaCo2 cells. B. Original dot-plots (PI/Annexin V) of a representative experiment demonstrating no significant difference between control and transfected cells.

Apoptosis was evidenced from Annexin V and propidium iodide binding. As illustrated in Fig. 5A, B, chorein significantly enhanced the percentage of ZF rhabdomyosarcoma cells in early or late apoptosis. In contrast, chorein silencing of Caco2 cells (Suppl. Fig. 1A), which express only low levels of chorein (demonstrated in Fig. 1A) had almost no effect on apoptosis of these cells (Fig. 6A,B).

Additional experiments addressed the effect of separate and combined chorein silencing and treatment with the cytotoxic drug Doxorubicin (500 nM). As shown in Suppl. Fig. 3, both, chorein silencing and doxorubicin treatment are followed by triggering of apoptosis. However, the combined treatment with chorein silencing and doxorubicin did not lead to further significant increase of apoptosis (Suppl. Fig. 3A middle). Chorein silencing did not significantly modify FITC dextran uptake (Suppl. Fig. 4).

## DISCUSSION

The present study discloses a completely novel regulator of tumor cell survival, i.e. chorein, the protein which is defective in patients with chorea-acanthocytosis. We show that chorein is expressed in several tumor cell lines with particularly high expression in ZF rhabdomyosarcoma cells. We further show that survival of those cells, but not of colon carcinoma cells with low levels of chorein expression is highly sensitive to the presence of chorein.

The present study further sheds some light on the mechanisms accounting for the impact of chorein on tumor cell survival. Chorein interacts with PI3K [2, 3] and chorein silencing decreases the phosphorylation of the PI3K-subunit-p85 in ZF rhabdomyosarcoma cells. The effect of chorein on PI3K is an example of “protein” addiction, whereby sudden abrogation of an anti-apoptotic protein causes apoptosis due to activation of caspases [16-19]

PI3K signaling counteracts apoptosis of a wide variety of cells including cancer cells [20-34] and neurons [35-38]. The present observations further reveal that chorein silencing stimulates the transcription of Bax, which is a powerful stimulator of apoptosis [39]. Chorein silencing further inhibits both, the transcription and protein expression of BCL-2, a potent inhibitor of apoptosis [40, 41]. The present study did not address the mechanisms accounting for altered BCL-2 and Bax expression following chorein silencing. At least in theory, the effect may again involve PI3K signaling, which leads to inhibition of FOXO transcription factors, powerful regulators of BCL-2 and Bax expression and activity [42-44]. Bax and BCL-2 are effective by influencing mitochondrial potential [45]. Accordingly, chorein silencing triggers mitochondrial depolarization. The stimulation of apoptosis by chorein silencing further involves activation of caspases, apoptosis executing enzymes [46-54].

Besides its effect on cell survival, chorein stabilizes the cortical actin filament network [1] thus influencing cell shape, exocytosis, membrane blebbing and receptor function [1, 55-63]. It remains to be shown whether the effect of chorein on actin cytoskeleton impacts on cell motility.

In view of the extra-cerebral expression and functions of chorein, chorea-acanthocytosis is a systemic disease presumably affecting a wide variety of functions. Chorein expression is particularly high in testis, kidney, spleen and brain [13]. Apparently, function and survival of neurons and skeletal muscle cells are particularly dependent on chorein [5, 10]. According to the present observations, some tumor cells similarly depend on chorein expression for survival. A more detailed analysis is expected to disclose chorein sensitive functions and survival of further cell types.

In view of the present observations, the lack of chorein in chorea-acanthocytosis may confer some protection against the development of specific malignancies. It is tempting to speculate that pharmacological interference with the interaction between chorein and PI3K may enhance the susceptibility of chorein rich tumor cells to treatment. PI3K inhibitors are effective in the treatment of malignancy but create considerable side effects due to disruption of the multiple PI3K dependent cellular functions [27, 64]. Chorein inhibition would not be expected to completely disrupt PIK3 signaling and compromise function and survival only of those cells expressing high levels of chorein and depending on chorein sensitive PI3K signaling. Along those lines complete lack of chorein in patients suffering from chorea-acanthocytosis requires decades to generate clinically relevant disorders [4-9].

In conclusion, the present observations reveal a novel function of chorein, i.e. the stimulation of tumor cell survival. Future studies will be required to define the impact of chorein expression on malignancy and therapy resistance of defined malignancies.

## MATERIALS AND METHODS

### Cells

Chorein transcript levels were determined in ZF rhabdomyosarcoma cells (established at the Children's Hospital Tuebingen from a multifocal, alveolar rhabdomyosarcoma of an eight year old girl) UW228-3 medulloblastoma cells (kindly provided by S Pfister, DKFZ Heidelberg) CaCo2 (ATCC, USA.) colon carcinoma cells, 451Lu melanoma (kindly provided by T. Sinnberg, Dermatology Tübingen), HF normal dermal skin fibroblasts (Promocell) and MCF-7 breast carcinoma cells (from ATCC, USA). All cells were seeded at 3×10^5^ cells/ml and grown for 48 h before RNA isolation in DMEM high glucose medium (Gibco) containing 10% FBS and 1% penicillin/streptomycin.

### Silencing of chorein

ZF rhabdomyosarcoma cells and CaCo2 cells were grown in DMEM high glucose medium (Gibco) containing 10% fetal bovine serum and supplemented with 1% penicillin/streptomycin under standard culture conditions (37°C, 5% CO_2_). Cells (1 × 10^5^) were seeded in 6 well plates 24 h before transfection. The cells were subsequently transfected with validated siRNA for VPS13A (chorein) (ID# s23342, Ambion, Darmstadt, Germany) or with a negative control siRNA (ID#4390843, Ambion) using siPORT amine transfection agent (Ambion) according to the manufacturer's protocol. The efficiency of silencing was checked by RT-PCR (Fig. 1B, Suppl. Fig. 1A, and Suppl. Fig. 2A).

### Cytostatic treatment

Not silenced and chorein silenced (48 h) ZF cells were treated with 500 nM Doxorubicin (Sigma-Aldrich, Germany) for 24 h under standard culture conditions (37°C, 5% CO_2_).

### RT-PCR

To determine transcript levels, total RNA was isolated 24 h, 48 h and 72 h after transfection using the Trifast Reagent (Peqlab, Erlangen, Germany). Two μg RNA was reverse-transcribed using oligo(dT)_12-18_ primers and GoScript Reverse Transcriptase Kit (Promega) according to the manufacturer's protocol. Quantitative real-time PCR was performed with the BioRad iCycler iQ^TM^ Real-Time PCR Detection System (Bio-Rad Laboratories) using GoTaq Sybr Green Master Mix (Promega). The reaction was applied in a final volume of 20 μl containing 2 μl of cDNA under following conditions: an initial incubation at 95°C for 5 min, 40 cycles at 95°C for 15 s, 59°C for 20 s and 72°C for 30 s. Specificity of the PCR products was verified by melting curve analysis. The subsequent primers were used (5′→3′ orientation):

VPS13A fw: AGTGGGACGACGTCTGTACAC
VPS13A rev:AGTTCTCATCTTCTGGCTTCAG
BCL-2 fw: TGGATGACTGAGTACCTGAACCG
BCL-2 rev: TGAGCAGAGTCTTCAGAGACAGC
BAX fw: ACTGGACAGTAACATGGAGCTG
BAX rev: AGCCCATGATGGTTCTGATCAG
GAPDH fw: TGAGTACGTCGTGGAGTCCACTG
GAPDH rev: GGTGCTAAGCAGTTGGTGGTG

The mRNA expression levels of the respective genes were normalized to the expression levels of GAPDH in the same cDNA sample. Relative quantification of gene expression was calculated according to the ΔΔCt method.

### Western blotting

To quantify protein abundance, cells were washed twice with ice cold PBS and suspended in 200 μl ice-cold RIPA lysis buffer (Thermo Fisher Scientific, USA) containing Halt Protease and Halt Phosphatase Inhibitor Cocktail (Thermo Fisher Scientific, USA). The protein concentration was determined using the Bradford assay (BioRad, München, Germany). Thirty μg of protein were solubilized in sample buffer at 95°C for 5 min. The samples for chorein detection were separated by NuPAGE® Novex® 3–8% Tris-Acetate gels (Life Technologies, Thermo Fisher Scientific, USA) in a Tricine-Tris buffer. For the remaining proteins a 10% SDS-PAGE in a Glycine-Tris buffer was used. After separation proteins were electro-transferred onto PVDF membranes for 90 min and blocked with 5% non-fat milk or 5% BSA in TBS-0.10% Tween 20 at room temperature for 1 h. Then, the membranes were incubated with appropriate primary antibodies: anti-PI-3K p85 antibody (1:1000, Cell Signaling), anti-phospho-PI-3K p85 (Tyr458)/p55 (Tyr199) antibody (1:1000, Cell Signaling), anti BCL-2 antibody (1:1000, Cell Signaling), anti-VPS13A-antibody (1:1000, Sigma-Aldrich, Germany) and anti-GAPDH antibody (1:2000, Cell Signaling) at 4°C overnight. After washing (TBST), the blots were incubated with secondary anti-rabbit (1:2000, Cell Signaling) antibody for 1 h at room temperature. Antibody binding was detected after additional washes (TBST) with the ECL detection reagent (Amersham, Freiburg, Germany) and quantified with Quantity One Software (BioRad, München, Germany). To assign the right protein size we used Protein-Marker V (Peqlab, Erlangen, Germany) and HiMark™ Pre-stained Protein Standard (Life Technologies, USA).

### FACS analysis

To determine the apoptotic response we used the Annexin V Apoptosis Detection Kit (MabTag, Germany). After silencing cells were harvested from the 6 well plates by treatment with trypsin-EDTA (Sigma-Aldrich, Germany) for 10 min and washed once with cell culture medium. After a further wash with PBS and centrifugation at 1600 RPM for 3 min at RT cells were suspended in 100 μl binding buffer containing Annexin V-FITC and propidium iodide and incubated for 20 min in the dark at RT. In the following the cells were washed once, resuspended in 200 μl binding buffer and measured immediately using the BD FACS Calibur (BD Biosciences, USA). For Doxorubicin treated cells TO-PRO®-3 Iodide (1:1000, Life Technologies, USA) was used instead of propidium iodide.

Active caspase-3 was estimated utilizing CaspGlow Fluorescein Active Caspase-3 Staining Kit (BioVision, USA) according to the manufacturer's instructions. After detaching and one wash with cell culture medium cells were suspended in 300 μl of complete DMEM (Gibco, USA) including 1 μl of FITC conjugated inhibitor of active Caspase-3 (FITC-DEVD-FMK). After 1h incubation and two washes with supplied wash buffer, cells were resuspended in 300 μl of wash buffer and analyzed by flow cytometry (BD FACS Calibur, BD Biosciences, USA).

Depolarization of the outer mitochondrial membrane was measured by incubating 10^5^ cells in 10 ng/ml JC9 (Life Technologies, USA) in the dark for 10 min at 37^o^ C. The cells were washed once in PBS at 1600 g for 3 min. A potential-dependent shift of fluorescence emission from 525 nm (FL1) to 590 nm (FL2) in the mitochondria was measured immediately by flow cytometry (BD FACS Calibur, BD Biosciences, USA).

FITC-dextran uptake was measured after 2 hours incubation with FITC-conjugated dextran (500 μg/ml, Sigma-Aldrich, Germany) in serum free DMEM medium at 37°C and on ice (background). The intake was stopped by adding ice-cold PBS to the cells. After three washes with ice-cold PBS, cells were re-suspended in 200 μl of PBS and analyzed by flow cytometry (BD FACS Calibur, BD Biosciences, USA). For the calculation, mean fluorescence values of the samples incubated on ice were subtracted from corresponding values incubated at 37°C.

### Confocal laser scanning microscopy

For chorein staining, ZF and CaCo2 cells were cultured on glass cover slips for 24 h. After washing twice with PBS, cells were fixed with 4% PFA for 15 min on RT and then permeabilized with 0,03% *Triton*-X100 for 10 min. After blocking with 3% BSA in PBST cells were incubated at 4°C overnight with anti-VPS13A-antibody (1:300, Sigma-Aldrich, Germany). The cells were rinsed three times with PBST and incubated with secondary goat anti-rabbit CF^TM^ 488 antibody (1:300, Sigma-Aldrich, Germany) for 2 h at room temperature. Additional cells were incubated 30 min in the dark with rhodamine-phalloidin (1:200, Life Technologies, USA) for F-actin staining and with DRAQ-5 dye (1:3000, Biostatus, Leicestershire, UK) for nuclei staining. After three washing steps all slides and coverslips were mounted with ProLong Gold antifade reagent (Life Technologies, USA). Images were subsequently taken on a Zeiss LSM 5 EXCITER confocal laser scanning microscope (Carl Zeiss, Germany) with a water immersion Plan-Neofluar 63/1.3 NA DIC.

### Statistics

Data are expressed as arithmetic means ± SEM. Statistical analysis was made by unpaired t-test. A p<0.05 value was considered statistically significant.

## SUPPLEMENTARY MATERIALS, FIGURES


