# Supplementary material for: Chorein addiction in VPS13A overexpressing rhabdomyosarcoma cells
Source: Oncotarget. 2015 Mar 14;6(12):10309–19. doi: 10.18632/oncotarget.3582 (PMC4496357; doi:10.18632/oncotarget.3582)
Supplement: Supplementary file 1 [file oncotarget-06-10309-s001.pdf]

Chorein addiction in VPS13A overexpressing rhabdomyosarcoma cells

Supplementary Material

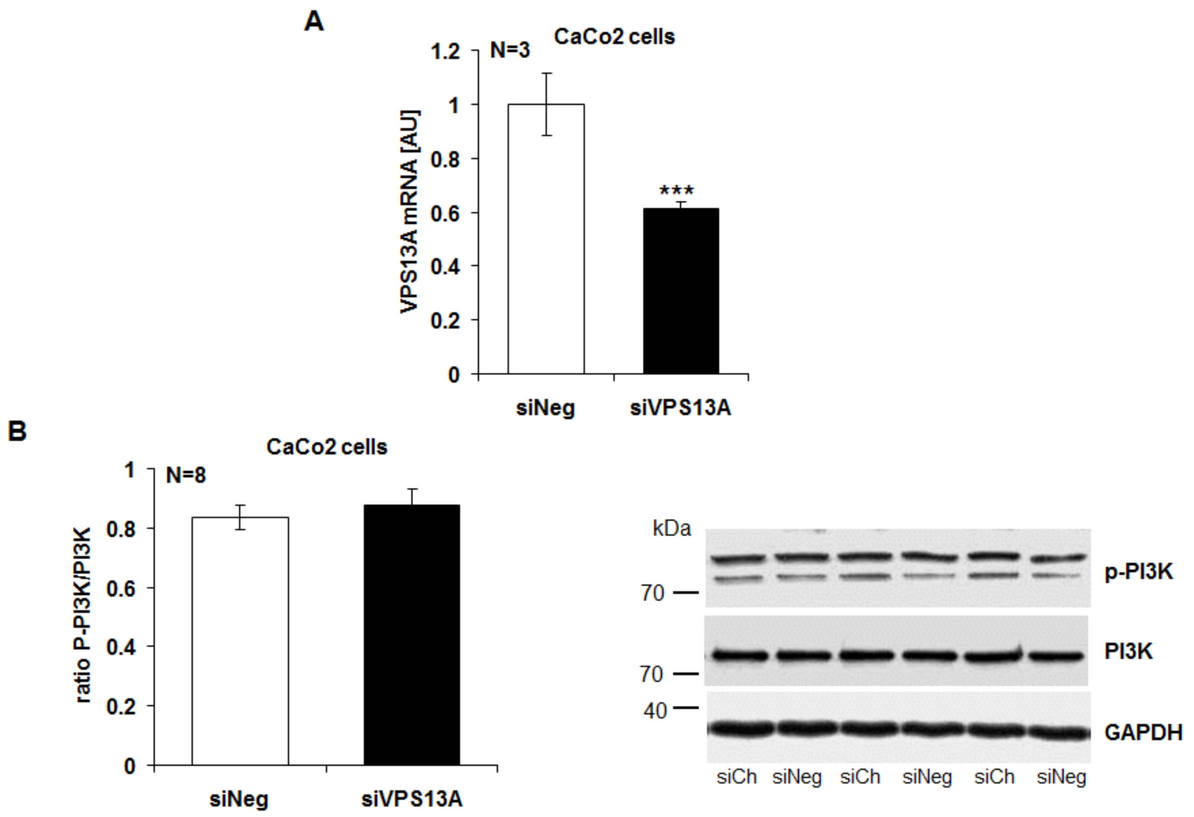

Supplemental Figure 1: Lack of effect on PI-3K p85 phosphorylation in CaCo2 cells after chorein silencing.

A. Chorein silencing efficiency of CaCo2 cells. Chorein (VPS13A) mRNA levels were analysed by quantitative real-time PCR. Bars indicate the mean values of $2^{-\Delta Ct}$ using GAPDH as housekeeping gene ± SEM from 3 independent experiments. *** (p< 0,001; unpaired t-test) significant difference to respective value of negative silenced control.

B. Left: arithmetic means ± SEM (n=8) of the ratio of phosphorylated PI-3K p85 subunit to total PI-3K (p-PI-3K/PI-3K ratio) in CaCo2 cells transfected with control siRNA (siNeg) and siRNA for chorein (siVPS13A). Right: representative original western blots showing the protein abundance of phosphorylated (p85) PI-3K, total PI-3K and respective GAPDH as loading control.

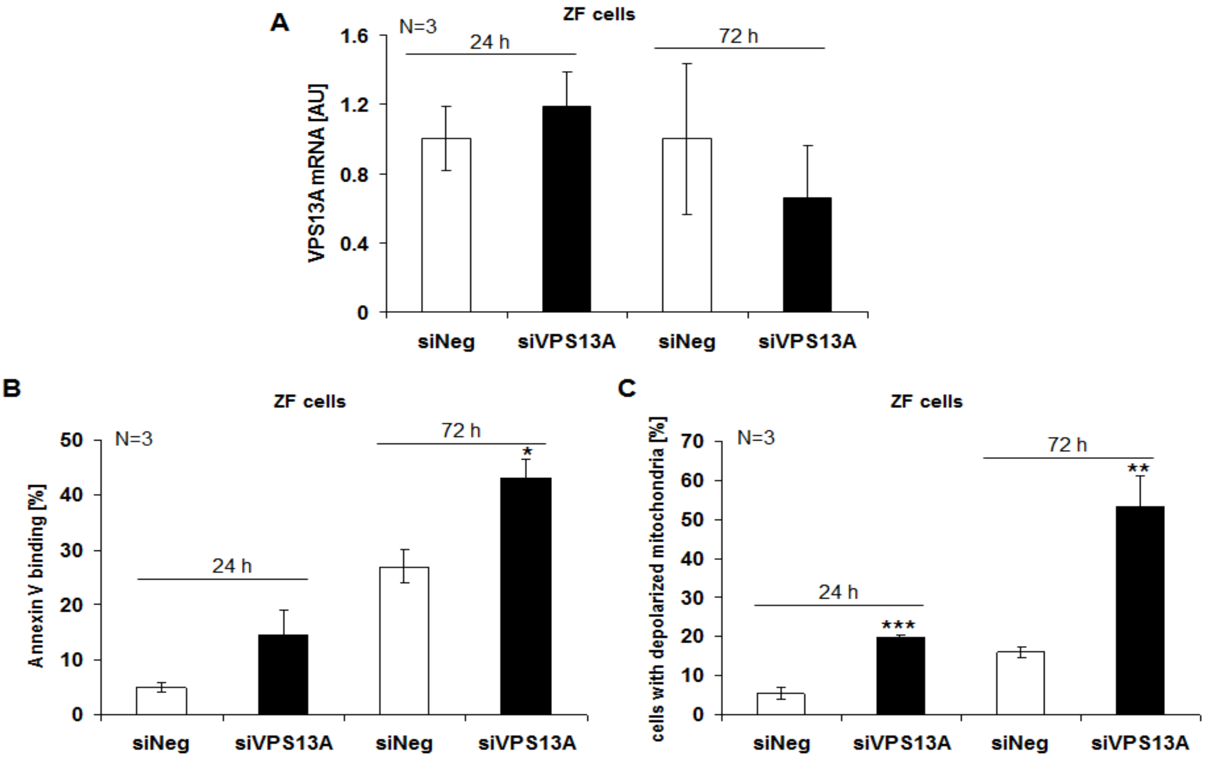

Supplemental Figure 2: Time dependend chorein silencing and apoptotic indicators in ZF rhabdomyosarcoma cells.

A. Chorein (VPS13A) mRNA levels in ZF cells were analysed by real-time PCR after 24h and 72h transfection. Bars indicate the mean values of $2^{-\Delta Ct}$ using GAPDH as housekeeping gene ± SEM from n=3 independent experiments. Chorein silencing was not sufficient after 24h (left bars) and 72h (right bars) transfection of ZF cells. No significant difference to the respective value of negative silenced control.

B. To indicate apoptosis after 24h and 72h transfection ZF cells were stained with FITC conjugated Annexin V. Presented are arithmetic means ± SEM (n=3) of Annexin V positive cells transfected with negative control siRNA (siNeg) or with siRNA for chorein (siVPS13A). *significant difference ($p<0.05$; unpaired t-test) to the negative silenced cells (siNeg) for 72h.

C. Arithmetic means ± SEM (n=3) of mitochondrial depolarization measured by FACS in ZF cells transfected for 24h and 72h with negative control siRNA (siNeg) or siRNA for chorein (siVPS13A).*** ($p<0.001$, unpaired t-test), and ** indicate significant difference ($p<0.01$, unpaired t-test) to the negative silenced cells (siNeg).

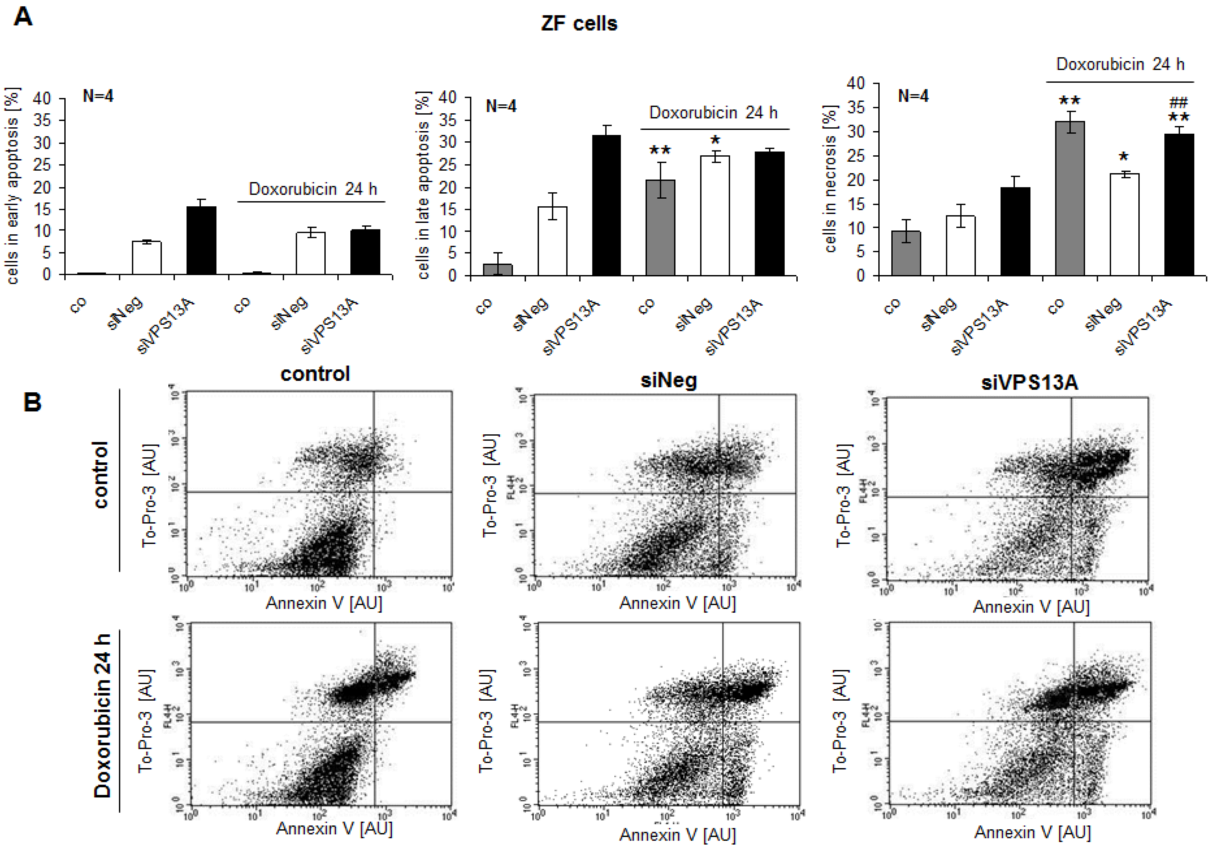

Supplemental Figure 3: Influence of cytostatic treatment and chorein silencing on cell death of ZF rhabdomyosarcoma cells.

A. ZF cells were silenced (48h) and treated or not with Doxorubicin (500 nM) for 24 h. Shown are arithmetic means (± SEM, n = 4) of early apoptotic (left), late apoptotic (middle) and necrotic (right) cells in percent. Grey bars indicate nonsilenced control, white bars indicate cells silenced with negative control siRNA (siNeg) and black bars show chorein silenced cells (siVPS13A). ** ($p<0.01$), * ($p<0.05$) indicate statistically significant difference from respective value without Doxorubicin exposure, ## ($p<0.01$) indicates significant difference to the negative silenced cells (siNeg) treated with Doxorubicin (unpaired t-test).

B. Original dot-plots (PI/Annexin V) of a representative experiment. Upper panel: without Doxorubicin exposure, lower panel after 24h Doxorubicin treatment.

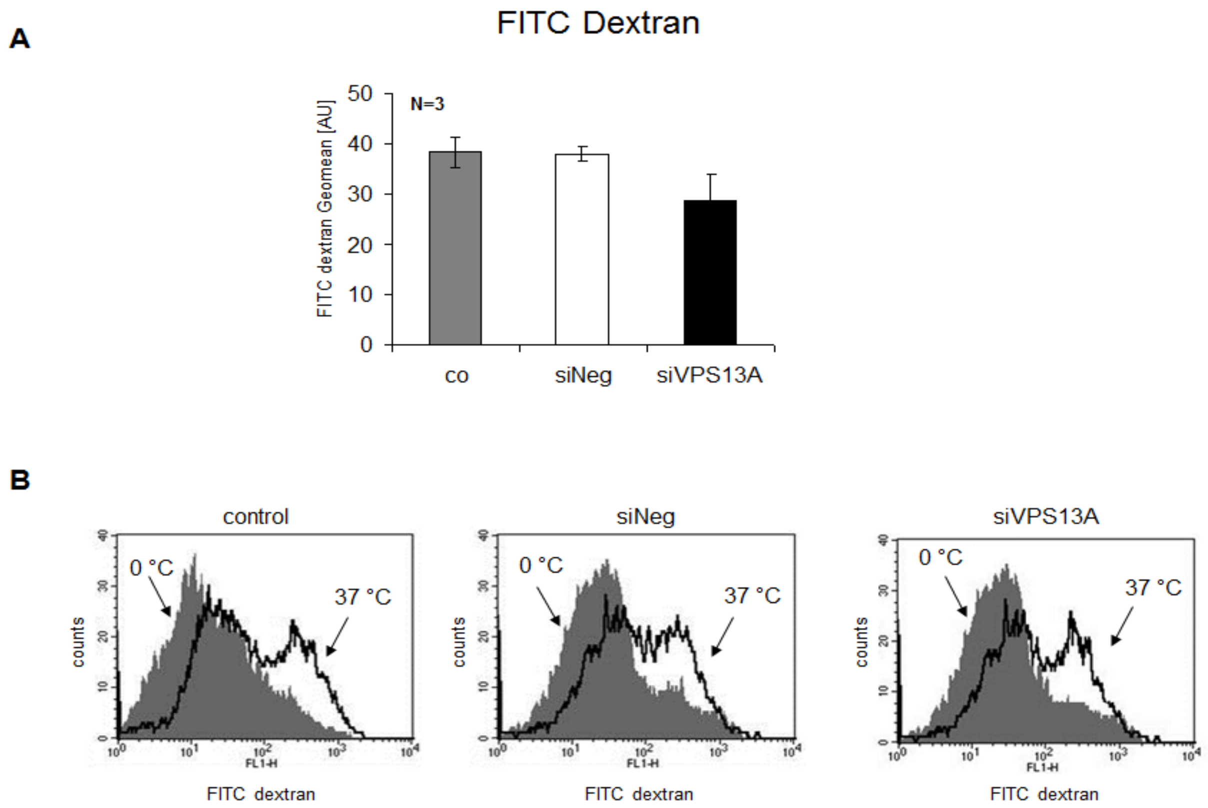

Supplemental Figure 4: Effect on FITC-dextran uptake of chorein silenced ZF rhabdomyosarcoma cells.

A. Silenced (48h) and not silenced (control) ZF cells were treated with FITC-dextran (500 µg/ml) and incubated for 2h at 37°C and on ice (background). Shown are arithmetic means (± SEM, n = 3, of the fluorescence intensity). The chorein silenced cells tended to take up less FITC-dextran , than negative silenced cells (siNeg), a difference, however, not reaching significance (unpaired t-test).

B. Original representative histograms of FITC-dextran fluorescence (cell events/FITC dextran) from control ZF cells and ZF cells transfected with negative siRNA (siNeg) or siRNA for chorein (siVPS13A). Grey: fluorescence after incubation on ice, black: fluorescence after incubation at 37°C.
